# Additively Manufactured Dragonfly-Inspired Wings for Bio-Faithful Flapping MAV Development

**DOI:** 10.3390/biomimetics10120849

**Published:** 2025-12-18

**Authors:** Emilia Georgiana Prisăcariu, Oana Dumitrescu, Sergiu Strătilă, Mihail Sima, Claudia Săvescu, Iulian Vlăducă, Cleopatra Cuciumita

**Affiliations:** Romanian Research and Development Institute for Gas Turbines COMOTI, 061126 Bucharest, Romania; sergiu.stratila@comoti.ro (S.S.); mihail.sima@comoti.ro (M.S.); claudia.borzea@comoti.ro (C.S.); iulian.vladuca@comoti.ro (I.V.); cleopatra.cuciumita@comoti.ro (C.C.)

**Keywords:** dragonfly wing morphology, biomimetic micro air vehicles, venation architecture, flapping-wing aerodynamics, Additive Manufacturing

## Abstract

This work presents a first-iteration bio-faithful dragonfly-inspired wing designed for future flapping micro air vehicle (MAV) applications. Using high-resolution imaging, the natural venation pattern of fore- and hindwings was reconstructed in CAD and reproduced through high-precision stereolithography at 1:1 and 3:1 scale. The printed polymeric wings successfully preserved the anisotropic stiffness distribution of the biological structure, enabling realistic bending and torsional responses. Modal analysis and dynamic testing confirmed that the lightweight designs operate within the biologically relevant 20–40 Hz range and that geometry and material choices allow predictable tuning of natural frequencies. Preliminary aerodynamic estimates captured the characteristic anti-phase lift behavior of four-wing flapping, while schlieren and infrared thermography demonstrated that heat dispersion and flow features follow the vein-driven structural pathways of the printed wings. Together, these results validate the feasibility and functional relevance of bio-faithful venation architectures and establish a solid foundation for future iterations incorporating membranes, full kinematic actuation, and higher-fidelity aeroelastic modeling.

## 1. Introduction

Bio-inspired micro air vehicles (BMAVs) have emerged as a compelling alternative to conventional fixed-wing or rotorcraft platforms for operations in low-Reynolds-number and cluttered environments. Their appeal stems from the unique aerodynamic benefits of flapping wings, which can generate lift and thrust efficiently at small scales and under flow conditions where steady aerodynamic approaches lose effectiveness. Early BMAV research explored simplified flapping mechanisms with symmetric wing pairs, focusing primarily on generating hover-capable prototypes and understanding the fundamental kinematics of small-scale flight (e.g., robotic hummingbirds, insect-scale resonant wings, and thorax-driven mechanisms) [1,2,3,4,5,6]. These efforts demonstrated that flapping platforms can outperform conventional aircraft in maneuverability, gust tolerance, and energy efficiency at small scales.

In recent years, attention has shifted toward biologically faithful geometries and kinematics, as numerous studies have shown that morphology plays a decisive role in aerodynamic performance. Dragonfly-inspired designs have been particularly influential. Investigations into corrugated wings, asymmetric flapping, and tandem wing phasing have revealed potential improvements in lift generation, hover stability, and aerodynamic efficiency [7,8,9]. Experimental and computational work has further highlighted the importance of multi-wing interaction, flexible membranes, passive deformation, and structural anisotropy for improving performance in BMAV configurations [10,11]. These findings have inspired a new generation of MAVs that incorporate bio-accurate vein–membrane structures, compliant hinge regions, and kinematically adaptive actuation strategies.

Parallel to biological and aerodynamic insights, technological developments in additive manufacturing, micro-actuation, and high-speed optical diagnostics have substantially expanded the design space available to BMAV researchers. Recent studies have demonstrated the feasibility of producing high-fidelity flapping wings using polymer-based 3D printing, biodegradable composites, and hybrid membrane–vein assemblies [10,12,13,14,15]. Numerical tools have likewise advanced: coupled Computational Fluid Dynamics–Finite Element Analysis (CFD–FEA) frameworks, Large-Eddy Simulation (LES)-based vortex analysis, and open-source unsteady solvers such as Ptera have enabled more accurate predictions of unsteady forces, wing deformation, and vortex interactions [16]. These advances support the transition from conceptual designs toward validated prototype demonstrators.

BMAV research has also attracted interest from the planetary exploration community. Studies investigating flapping-wing hover on Mars have shown that bio-inspired wings can generate sufficient aerodynamic force through appropriate scaling, kinematic tuning, and structural compliance adjustments [17,18,19]. These results suggest that highly optimized biological wing architectures (particularly those of dragonflies, which excel in low-Re maneuverability) may serve as promising templates for next-generation aerial scouts capable of operating in thin atmospheres.

The present work introduces an innovative dragonfly-inspired wing architecture that differs fundamentally from conventional biomimetic wings, which typically rely on simplified planform geometries, idealized corrugations, or uniformly flexible membranes. By reconstructing the full venation network at 1:1 biological scale and manufacturing it with high-resolution stereolithography, this study provides a structurally faithful wing skeleton that preserves the natural stiffness gradients inherent to Odonata wings. The enlarged 3:1 experimental model further enables high-resolution schlieren, IR thermography, and dynamic testing that are not feasible at the true insect scale. Together, these prototypes establish a reproducible platform for aeroelastic, structural, and actuation studies, representing a significant step toward physically realizable bio-inspired flapping-wing MAVs.

Motivated by these developments, the present work aims to integrate bio-faithful morphology, structurally optimized wing architectures, high-speed experimental validation, and numerical modeling into a unified framework for developing a dragonfly-inspired flapping system. Rather than focusing solely on simplified or idealized wing shapes, this study emphasizes geometries and kinematic features derived directly from natural dragonfly wings, supported by additive manufacturing and multimodal optical testing. By combining structural characterization, aerodynamic modeling, and high-fidelity flow visualization, this work contributes to the ongoing effort to bridge biological inspiration with engineering implementation in the BMAV domain.

## 2. Biological Inspiration and Theoretical Background

### 2.1. Dragonfly Flight Mechanics (LEV, Clap-and-Fling, Stroke-Plane Geometry)

**Figure 1:**
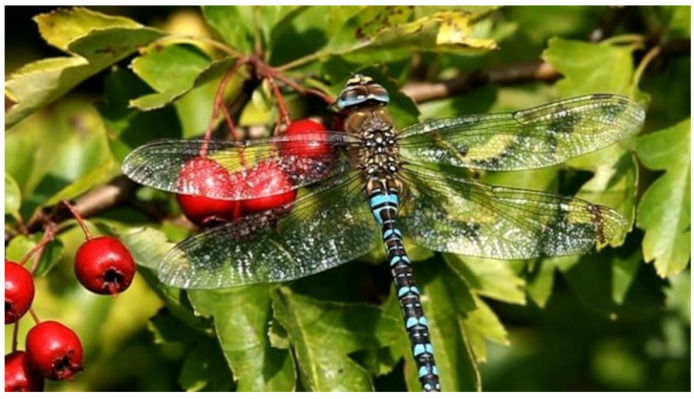
In-flight dragonfly (credits: National Geographic) [25].

Dragonflies exhibit some of the most refined aerodynamic strategies among flying insects, combining specialized morphology with complex wing kinematics to achieve exceptional maneuverability. Their two pairs of wings operate independently, enabling unique phasing patterns that modulate lift, thrust, and control authority during different flight modes. Classic entomological studies such as [20] demonstrated that forewing–hindwing phase relationships vary dynamically during turning, hovering, and acceleration, directly influencing vortex development and aerodynamic load distribution. High-speed flow-visualization work by Thomas et al. [21] revealed that dragonflies manipulate stroke amplitude, angle of attack, and wing phasing to control wake topology and stabilize leading-edge vortices. From a morphological perspective, the corrugated cross-sections and vein–membrane architecture of dragonfly wings, highlighted in Wootton’s functional morphology analyses [22,23], contribute to passive deformation that enhances lift and delays stall. More recent biological overviews, including Bomphrey et al. [24], have consolidated these insights by showing how structural anisotropy, wing twist, and mass distribution synergistically facilitate agile flight. Figure 1 displays an airborne dragonfly.

Dragonfly aerodynamic force production relies on several unsteady mechanisms. Stabilized leading-edge vortices (LEVs), dynamic stall suppression, and pronounced rotational lift during pronation and supination have been documented extensively in experimental and computational studies [26,27,28].

Additional work on forewing–hindwing vortex interactions [29,30] shows that varying phase offsets can either enhance lift through constructive vortex merging or reduce energetic cost by distributing aerodynamic loads across both wing pairs. Morphometric and kinematic analyses from Sun, Gong, and Huang [31] and Noda et al. [32] further highlight how the interplay between geometry and motion governs the formation, strength, and trajectory of vortical structures during different flight modes. Together, these studies underscore why dragonflies provide such a powerful biological model for the design of biomimetic micro air vehicles.

**Figure 2:**
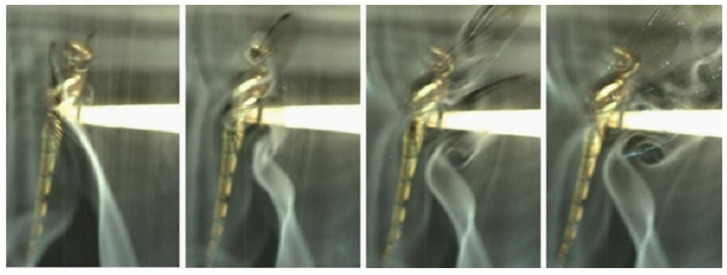
Vortex growth and shedding at the first downstroke of an in-phase flapping dragonfly [30].

Dragonflies are exceptionally quick and agile flyers, capable of rapidly changing direction and transitioning between hovering and forward flight. Their high power-to-weight ratio, partly due to the low aspect ratio of their wings, contributes significantly to their maneuverability. Additionally, their ability to rotate their wings about multiple axes enhances aerodynamic control, making them among the fastest and most agile flying insects in the world [14]. The complex structure of dragonfly wings plays a central role in enabling these capabilities [11,15,33]. Bomphrey et al. [24] investigated the aerodynamic performance of real dragonfly wings by reconstructing detailed 3D geometries and comparing them with artificial wings through computational fluid dynamics (CFD) and particle image velocimetry (PIV) analyses. Their simulations across various speeds and angles of attack revealed that the forewing operates within a region of positive pressure generated by the hindwing, which reduces its drag, while the hindwing experiences increased drag due to the influence of the forewing. Similarly, Hefler et al. [30] conducted PIV measurements of airflow around a tethered live dragonfly, focusing on the aerodynamic effects of wing phasing. Their smoke visualizations (Figure 2) showed that during in-phase flapping, a leading-edge vortex (LEV) forms on the forewing, while a trailing-edge starting vortex develops on the hindwing during the first downstroke. They proposed that dragonflies employ in-phase flapping during takeoff to generate strong downward momentum—a conclusion supported by Alexander [20]. In this configuration, the tandem wings effectively function as a single large wing, producing one LEV and one trailing-edge starting vortex on the forewing.

**Figure 3:**
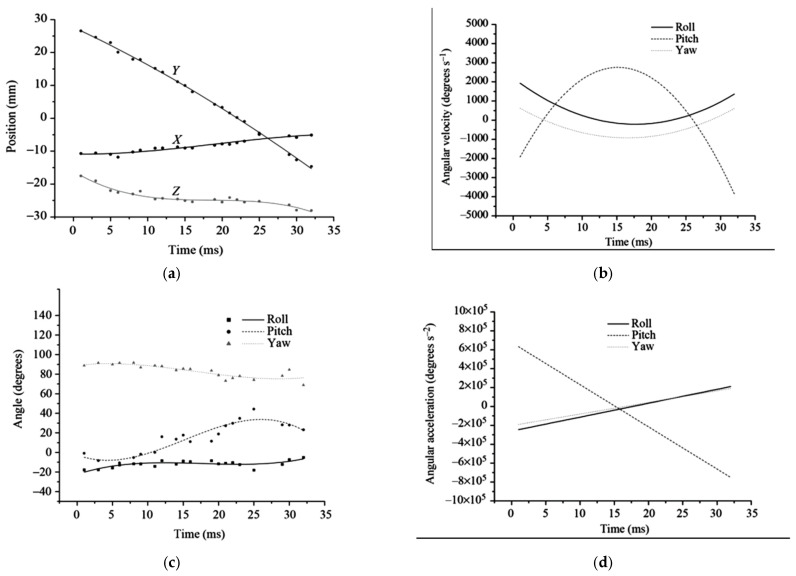
The measured flight (**a**) trajectory, (**b**) velocity, and (**c**,**d**) acceleration of turning maneuvers in dragonfly [31].

Hovering is a flight mode commonly used by dragonflies when targeting prey. During hovering, the dragonfly maintains a stationary position over a reference point on the ground, making only subtle adjustments in body posture to capture prey [34]. The wings operate mainly within an inclined stroke plane [35,36], and the forewings typically beat 180° out of phase with the hindwings [37,38], although both wing pairs flap at the same frequency. This counter-stroking pattern generates just enough lift to balance the dragonfly’s weight while minimizing power expenditure, allowing efficient energy conservation during stationary flight [39]. In contrast, during acceleration, dragonflies switch to an in-phase flapping pattern, with a 0° phase difference between forewings and hindwings [21]. This configuration enhances both lift and thrust production, enabling rapid acceleration to speeds of up to 13.4 m/s [40]. However, due to the high-power demand and short duration [41], this flight mode is less energy efficient. Consequently, dragonflies primarily use in-phase flapping during take-off or prey pursuit, where quick and powerful movement is essential [31]. Experimental results [31] reveal that during turning maneuvers, free-flying dragonflies exhibit an asymmetric angular velocity distribution over time (Figure 3b). Alongside changes in pitch angle (Figure 3), both roll and yaw angles increase at similar rates, demonstrating that the dragonfly executes a right turn with a rightward-tilted body and elevated head.

Experimental and numerical results show that synchronous flapping with high angles of attack maximizes lift during take-off, while increasing phase difference shifts the wing motion toward thrust-efficient modes [14].

Bioinspired flapping wing rotors incorporating passive rotation achieve high lift and propulsive efficiency through controlled vortex dynamics [42]. Tandem flapping wing mechanisms modeled after dragonflies generate about 50% more lift and improve flight stability compared to single wing pairs [7]. Further simulations reveal that wing spacing and relative positioning critically affect aerodynamic forces, efficiency, and leading-edge vortex formation, guiding the design of versatile, efficient biomimetic micro air vehicles [43]. Hu et al. [8] study presents the design and development of a dragonfly-inspired flapping-wing bionic air vehicle with three degrees of freedom. The design process draws from an analysis of dragonfly flight mechanics and body structure, leading to an optimized mechanism that replicates the characteristic “8”-shaped wing trajectory. Kinematic simulations show stable performance in three directions, while CFD analysis confirms efficient aerodynamic force generation at 5 m/s and 4 Hz. Wind tunnel experiments validate these results, with the prototype achieving an average lift of 3.62 N and thrust of 2.54 N. A second study numerically investigates the aerodynamic performance of a dragonfly-inspired cambered airfoil near the ground, comparing it to a conventional NACA4412 airfoil [44]. Using Unsteady Reynolds-Averaged Navier–Stokes (URANS) simulations, the study evaluates the influence of Reynolds number, Strouhal number, and ground clearance. At higher Reynolds numbers (Re = 5 × 10^4^), the NACA4412 outperforms the dragonfly airfoil at low frequencies, though performance converges at higher oscillation frequencies. At lower Reynolds numbers (Re = 5 × 10^3^), the dragonfly-inspired airfoil shows superior lift-to-drag ratios across all tested conditions, indicating its potential advantage in low-speed, near-ground MAV applications.

Another study on NACA 4412 compares the aerodynamic performance of a dragonfly-inspired airfoil and a conventional NACA4412 airfoil under varying Strouhal numbers (St), angles of attack (AoA), and Reynolds numbers (Re), using URANS simulations with the Transition SST model. [44] shows that at St = 0.2 and 0.6, the NACA4412 airfoil consistently produces higher lift coefficients (Cl) and lower drag (Cd) for positive AoA, while the dragonfly airfoil exhibits lower drag at negative AoA. Specifically, NACA4412 demonstrates 55% and 12% lower drag, and 63% and 54% higher lift, at St = 0.2 and 0.6, respectively. At Re = 5 × 10^4^, increasing St from 0.2 to 0.6 reduces the lift-to-drag ratio (Cl/Cd) significantly for both airfoils—more so for NACA4412, though it retains superior performance. This is attributed to a stronger leading-edge vortex (LEV) on the cambered NACA4412 airfoil. However, at Re = 5 × 10^3^, the dragonfly airfoil outperforms NACA4412 in Cl/Cd across all tested frequencies, due to its lower drag. These findings suggest that NACA4412 is more efficient at higher Re and lower St, while the dragonfly airfoil excels at low Re, making it more suitable for low-speed, bioinspired MAV applications [44].

Noda et al.’s study [32] analyzes how dragonfly wing kinematics influence flight performance in normal and escape modes using a validated CFD model. During escape flight, lift increases and thrust decreases, reducing aerodynamic efficiency by ~8% and causing a steeper ascent. The hindwing plays a greater role due to changes in vortex interactions, especially the LEV. While wing phase difference has a minor direct effect, it can help stabilize forces when combined with angle-of-attack adjustments. These insights support the development of agile, bioinspired micro air vehicles (MAVs).

**Figure 4:**
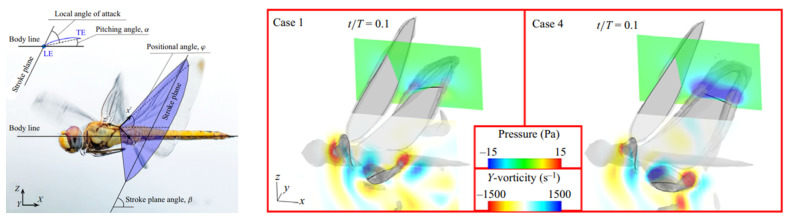
Kinematic and geometric angles of a dragonfly in flight: stroke plane (x′-y′) with stroke plane angle β, positional angle φ, and a pitching angle α [32] (**left**). Y-vorticity and pressure fields at 0.7 RHW illustrate how varying pitching angles affect LEV and TEV formation on the downstroking hindwing; iso-surfaces ($Q=5.0\times 10^{5}s^{-2}$) highlight vortex structures, with hidden flow regions beneath the wing [32] (**right**).

Figure 4 [32] displays vortex iso-surfaces based on the Q-criterion, along with pressure and vorticity at 0.7RHW of the hindwing during downstroke, comparing normal flight (case 1) and parametric case 4 at the moment of peak aerodynamic force modulation. The figure shows that a higher angle of attack during downstroke strengthens vortex structures along the hindwing edges, creating a negative pressure zone above the wing that increases lift. However, the clockwise pitching motion redistributes aerodynamic forces, converting thrust into drag, similar to the effect observed on the forewing.

**Figure 5:**
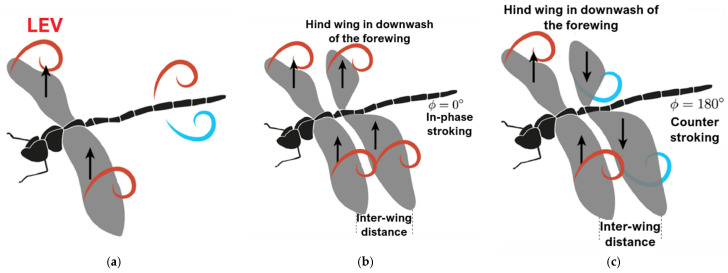
Schematic of vortex dynamics: (**a**) single wing, (**b**) tandem wing with flapping in-phase, and (**c**) tandem wings flapping out-of-phase, giving rise to wing-wing and wing-vortex interaction [45].

Quasi-steady theory underestimates the lift required for dragonfly flight, emphasizing the crucial role of unsteady aerodynamics in tandem-winged insects like dragonflies, whose fore- and hindwings flap independently. Unlike single-wing fliers, tandem wings generate complex wing-wing and wing-wake interactions that depend on wing phasing and spacing, particularly in in-phase and out-of-phase flapping modes. Figure 5 [45] highlights this key difference: single-wing fliers (Figure 5a) lack wake interactions, while tandem-wing configurations (Figure 5b,c) exhibit intricate aerodynamic phenomena due to wing and wake interference. This study concentrates on the two primary dragonfly wing phase modes—in-phase and out-of-phase flapping. To simulate these flapping wings, dynamic meshing techniques such as spring-based smoothing, dynamic layering, and local re-meshing are employed to efficiently adapt the mesh around moving wings, preserving accuracy and avoiding mesh errors during motion.

### 2.2. Morphological Features Relevant to MAV Design (Corrugations, Membranes, Veins)

**Figure 6:**
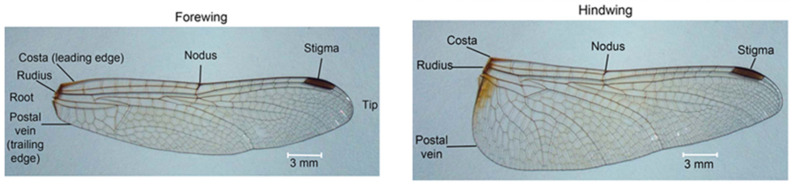
Real pattern of dragonfly wings [47].

The study by Lu et al. [46] reveals that the venation network in dragonfly wings is organized according to the golden ratio (φ ≈ 1.618) and the golden angle (≈137.5°). By analyzing 50 high-resolution wing images, the authors found that the most frequent inter-vein intersection angles cluster around 111° for both forewings and hindwings, which correlates with the golden-angle partition of 360° when three veins meet (137.5° for the largest angle and ~111.25° for the two equal smaller angles). They developed a “Golden-Ratio Partition” model to account for this angular distribution: the model shows that distorted polygonal venation cells (quadrilaterals, pentagons, hexagons) within the wing structure are partitioned by the golden rule (and low-order Fibonacci ratios) to optimize structural support and membrane reinforcement. Importantly, the golden-angle-dominated intersections concentrate near the wing’s trailing-edge and tip, regions where veins and membranes are thinnest and structural loading is highest, suggesting a biomechanical optimization for weight-bearing, flexibility, and aerodynamic function. Figure 6 presents the morphologically accurate pattern in dragonfly wings.

**Figure 7:**
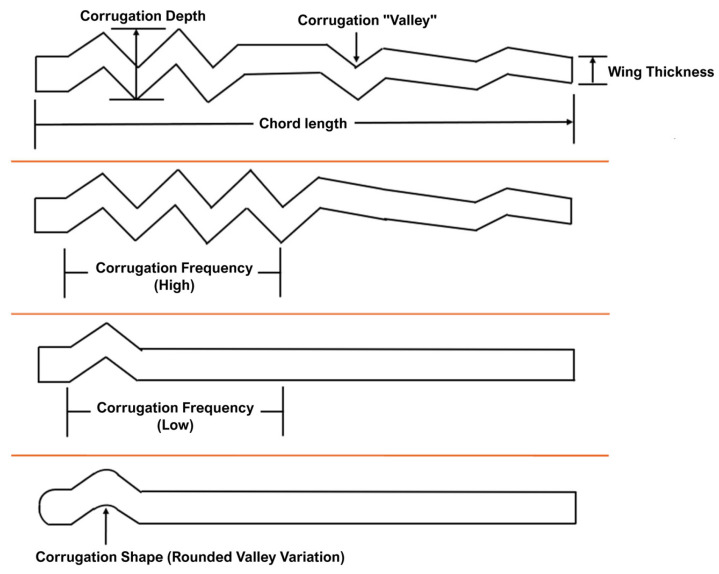
Corrugated wing geometry vs. detailed side profiles [48].

Building on the golden-ratio organization, the corrugated cross-section characteristic of dragonfly wings plays a crucial role in aerodynamic performance at low Reynolds numbers. Corrugations act as passive flow-control features, stabilizing the formation of a leading-edge vortex and delaying stall during high-angle-of-attack maneuvers. Studies on corrugated bio-inspired airfoils have repeatedly demonstrated superior lift-to-drag ratios, improved flow reattachment, and enhanced resistance to vortex breakdown compared to smooth, thin airfoils at comparable Reynolds numbers. These benefits extend directly to MAV design, where unsteady aerodynamic phenomena dominate, and conventional steady-airfoil behavior becomes inefficient. Figure 7 presents the corrugated profiles of a dragonfly wing, illustrating the variations in geometrical parameters with detailed side profiles, as described in [48].

The vein–membrane composite structure further enhances wing performance through its anisotropic stiffness distribution. Thick longitudinal veins provide structural support, while thinner transverse veins and membrane regions enable controlled torsion during flapping. This passive deformation allows the wing to dynamically adjust camber, twist, and chordwise curvature during each wingbeat, reducing energetic expenditure and improving LEV stability.

Finally, the membrane properties themselves are tuned for aerodynamic and structural performance. Dragonfly membranes are extremely thin (1–3 μm in many species), yet they exhibit high tensile strength and allow for micro-deformations that modulate surface curvature. These micro-deformations contribute subtly to load redistribution and LEV stability, particularly during the rapid deceleration phases of the wingbeat cycle. Studies comparing biological membranes with engineered polymeric films have confirmed that biomimetic membrane compliance improves aerodynamic performance by reducing peak stress and increasing flapping efficiency.

## 3. Materials and Models

### 3.1. Bio-Faithful Wing Geometry Acquisition

**Figure 8:**
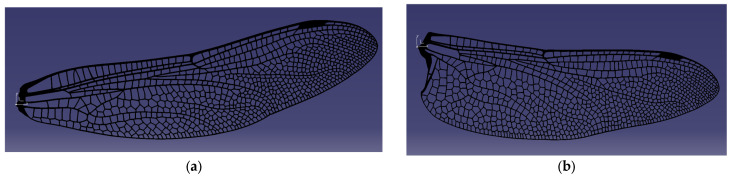
Bio-faithful CAD models of the forewing (**a**) and hindwing (**b**), designed in CATIA V5 with vein distributions matching the natural dragonfly morphology.

The first iteration of the wing design was carried out by preserving, as faithfully as possible, the actual morphology of the dragonfly fore- and hind-wings, which were digitally reconstructed and modeled in CAD at a 1:1 scale. High-resolution microscope and photographic references of the natural wing were used to reproduce the vein network, nodus position, and articulation geometry, ensuring that the structural layout captures the natural distribution of stiffness and mass. This approach allowed the creation of a geometrically accurate 3D model that retains the biologically optimized load-bearing pattern, where the dense vein lattice near the leading edge provides bending rigidity, while the trailing regions remain more compliant. The 3D geometries of the wings were extracted from the Australian Tiger Dragonfly species and designed in Catia V5. They are depicted in Figure 8a for the forewing and Figure 8b for the hindwing.

Such bio-inspired vein topology can be considered a valuable asset for BMAV integration, since it offers a naturally efficient trade-off between stiffness, flexibility, and mass, which can be directly exploited in future aeroelastic design iterations. After modeling, both the forewing and the hindwing were 3D-printed using photopolymer resin, resulting in a set of structural demonstrators that are geometrically true to the dragonfly wings, but at this stage produced with a flat profile to simplify manufacturing and testing. Although the printed models lack the natural camber and local thickness variation of the original wing, this first iteration enables straightforward optical and mechanical testing to identify the principal deformation modes and to calibrate the actuation system. The realistic vein distribution also facilitates the study of thermal propagation and deformation under heating, since the vein pattern acts as a conduction network and introduces anisotropic stiffness paths that influence both heat dispersion and vibration response.

### 3.2. Manufacturing Workflow

A first iteration of the wing fabrication method was achieved using additive manufacturing on a Formlabs Form 3+ [49] stereolithography printer, employing high-precision photopolymerization to reproduce the delicate nervure network of the dragonfly wing. The initial prototypes were printed at 1:1 scale, maintaining 100% fidelity in vein (nervure) placement and overall structural layout, followed by an enlarged 3:1 scale version to facilitate optical visualization and dynamic testing. Both variants currently feature a flat aerodynamic profile with a uniform wall thickness of 0.5 mm, representing early-stage structural validation models aimed at assessing stiffness and vibration response prior to membrane integration. The fabricated wings consist solely of the skeleton framework, produced in Formlabs Tough 2000 [50] photopolymer resin. The Form 3+ printing parameters were refined through ten iterative fabrication cycles to enhance surface resolution, ensure dimensional fidelity, and eliminate typical defects such as cupping, trapped resin, and uneven polymer flow. The optimal configuration was achieved by employing thin layer heights (50–100 µm), reduced peel speed, and extended laser exposure times, which together ensured complete curing of the fine vein geometry. Proper drainage channels were incorporated into the CAD model to promote resin flow and prevent vacuum pockets during printing. The parts were oriented at a 10–15° inclination relative to the build plate to minimize cupping and improve inter-layer adhesion, followed by isopropanol cleaning and 30 min UV post-curing to reach full mechanical strength. These optimized parameters resulted in high-precision, lightweight wing skeletons with excellent repeatability and a consistent 0.5 mm wall thickness, suitable for subsequent dynamic excitation, schlieren visualization, and quantitative analysis, as well as thermal dispersion tests.

**Figure 9:**
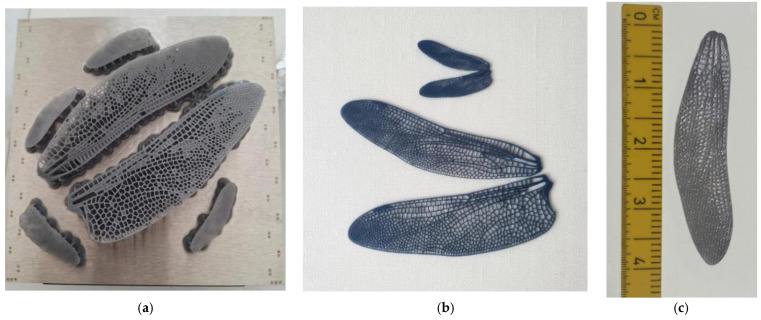
Tough 2000 resin 3D-printed flapping wings. (**a**) Resin structure on the printing pad; (**b**) post-processed 0.5 mm thick Tough 2000 resin; (**c**) 1:1 model of Tough 2000 nervures and silent cloth membrane next to measuring ruler (in cm).

**Figure 10:**
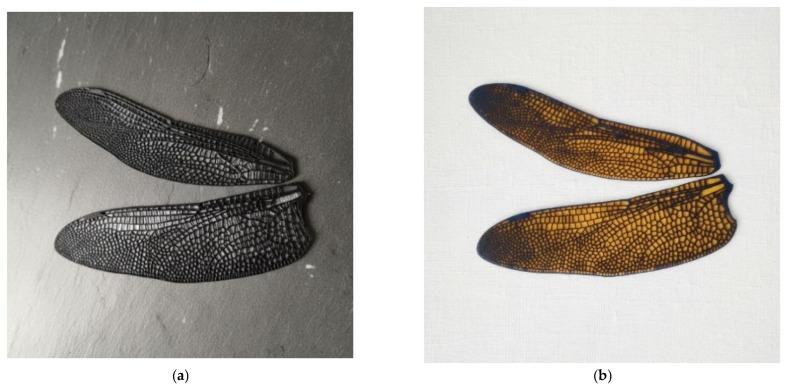
Illustration of models of the first-iteration 1:1 wing prototype integrating thin surface membranes. (**a**) bio-derived chitosan membrane [51], (**b**) Kapton^®^ [52] film bonded to the printed nervure framework.

The outcome of the first design and fabrication iterations is illustrated in Figure 9 and Figure 10, which present the 3D-printed wing prototypes produced during the optimization of the additive manufacturing process. The images show both the initial Form 3+ printing pad with multiple early-stage samples and the successive printed models used for parameter tuning and dimensional inspection. All wing prototypes were fabricated using a Formlabs Form 3+ stereolithography system with Tough 2000 resin. Printing was performed at a layer height of 50–100 μm, selected to balance geometric fidelity with build time for the fine venation network. The wings were oriented at 10–15° relative to the build plate to minimize cupping and ensure complete resin drainage, while custom support structures were added along the trailing edge to maintain dimensional stability. The nominal printer tolerance of ±0.1 mm for features above 0.5 mm was verified through caliper measurements of key vein thicknesses on multiple prints, confirming consistent reproduction of the CAD geometry.

After printing, the parts were washed in two consecutive baths of 99% isopropyl alcohol (IPA) for 10 min each using the Form Wash system, then air-dried for 20 min. Post-curing was performed in a Form Cure unit using 405 nm LED illumination at 60 °C for 30 min, ensuring complete polymer crosslinking and stable mechanical properties across all 1:1 and 3:1 prototypes. These printing and curing parameters were kept constant throughout the fabrication process to ensure high reproducibility and dimensional accuracy of the wing skeletons.

These early prints were essential for validating the fidelity of the CAD-to-print transfer, confirming the successful reproduction of the intricate nervure network and joint regions. The series of models demonstrates the progressive improvement in print quality achieved through adjustments in orientation, support geometry, and resin flow management. Each iteration contributed to refining the structural definition of the 1:1 and 3:1 scale wings, ultimately yielding a stable and repeatable printing configuration. The printed demonstrators thus provide tangible proof of the manufacturing feasibility and reproducibility of the bio-inspired wing structure, forming the foundation for the subsequent mechanical and optical testing phases. Both models in Figure 10 demonstrate compatibility between the Form 3+ printed skeleton and flexible membrane integration for future aero-elastic tests.

The first integration tests with chitosan and Kapton^®^ membranes highlighted the importance of selecting an appropriate flexible surface material for future composite wing designs. Membrane materials must satisfy several requirements: low density to avoid penalizing inertial loads, moderate in-plane stiffness to enable passive deformation and LEV stabilization, high fatigue resistance under cyclic flapping, strong adhesion to the 3D-printed venation network, and uniform thickness for repeatable aerodynamic behavior. Chitosan films offer high flexibility and favorable damping characteristics, making them suitable for reproducing the soft, compliant nature of biological membranes. Kapton^®^, by contrast, provides excellent thermal stability, mechanical durability, and dimensional consistency, useful for controlled laboratory testing. These early trials establish the baseline criteria that will guide membrane selection in upcoming composite wing iterations and inform the coupling between the vein skeleton and the aerodynamic surface.

### 3.3. Structural Modeling (FEA)

**Figure 11:**
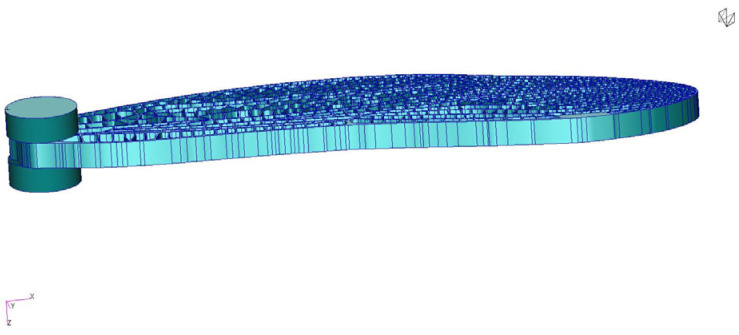
Representation of the experimental setup of the 1:1 scale wing, mounted between two ballistic gelatine pods.

Six configurations of the flapping-wing structure were analyzed using finite element modal simulation to evaluate the influence of material stiffness, vein thickness, and boundary conditions on natural frequencies and deformation modes: (a) Case 1: Tough 2000 resin, 0.5 mm veins, fixed root, exhibited a first bending mode at ≈55 Hz and a torsional mode at ≈189 Hz, closely matching the 1:1 experimental prototype and serving as the baseline for validation, (b) Case 2: Tough 2000 resin, 0.5 mm veins, mass-attached root, the added root mass slightly lowered the first mode to ≈45 Hz, highlighting the impact of attachment configuration on resonance behavior, (c) Case 3: Tough 2000 resin, 0.25 mm veins, fixed root, reducing the vein thickness by half significantly decreased stiffness, with the first mode dropping to ≈30 Hz, suggesting potential for resonance matching with low-frequency actuation, (d) Case 4: Tough 2000 resin, 0.25 mm veins, mass-attached root, combined geometric softening and inertial loading reduced the first mode further to ≈20 Hz, demonstrating the tunability of the design toward the biological 25–30 Hz flapping range, (e) Case 5: Inconel 718, 0.5 mm veins, mass-attached root, the metallic skeleton achieved a bending mode near ≈ 880 Hz and a torsional mode near 1.8 kHz, confirming high stiffness and relevance for hybrid wing structures requiring structural reinforcement, (f) Case 6: Inconel 718, 0.25 mm veins, fixed root, frequency values remained in the high-hundreds Hz range, indicating that material stiffness dominates over geometric variation in metallic configurations. The boundary conditions with the fixed degrees of freedom are presented in Figure 11.

The finite-element analyses used material properties consistent with manufacturer data for Tough 2000 resin and standard engineering references for Inconel 718. Tough 2000 was modeled with a density of 1200 kg/m^3^, Young’s modulus of 2.2 GPa, and a Poisson ratio of 0.35, representative of the post-cured polymer. Inconel 718 was assigned a density of 8190 kg/m^3^, Young’s modulus of 200 GPa, and a Poisson ratio of 0.29, corresponding to its conventional wrought mechanical properties. These values were applied uniformly across the corresponding cases to ensure consistent structural comparison between polymeric and metallic configurations.

**Figure 12:**
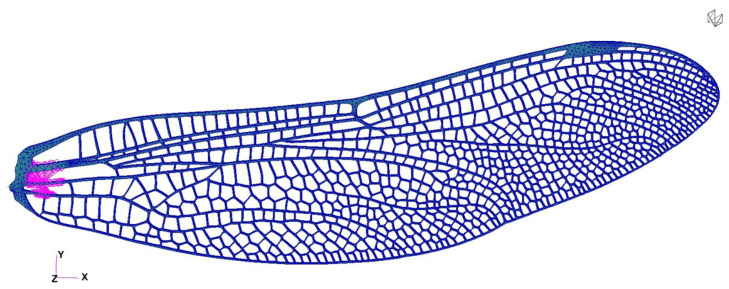
Boundary Conditions: fixed RBE2 nodes (from magenta region; all degrees of freedom are fixed).

It should be noted that the numerical baseline model (Case 1) represents the 3:1 scaled geometry, whereas the measured natural frequency of 24.7 Hz (experimentally tested between two ballistic gelatine pods—Figure 11) corresponds to the 1:1 printed prototype. Because natural frequencies scale approximately with 1/L for slender wing-like structures, the larger 3:1 geometry naturally exhibits a significantly higher bending-mode frequency (~55 Hz in a rigid support). Additional reductions in the experimental value arise from manufacturing tolerances, resin thickening, and clamping compliance in the physical test. This distinction explains the apparent discrepancy and confirms that each scale accurately reflects its intended function within the study. Figure 12 presents the fixed RBE2 nodes.

**Figure 13:**
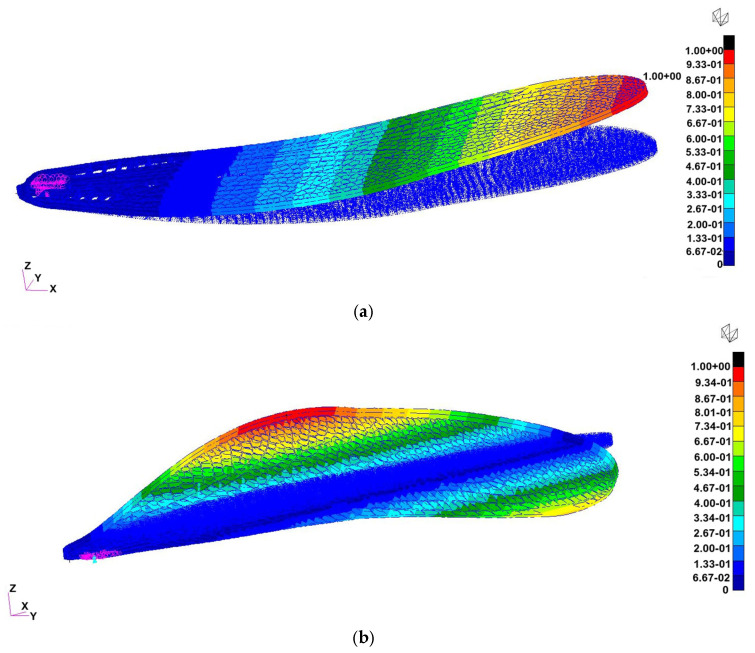
Normal-mode shapes for Case 1 (Tough 2000 resin, 0.5 mm veins, fixed root): (**a**) First bending mode at ≈55 Hz (left) (in this case, the wing was fixed in a rigid support), (**b**) first torsional mode at ≈189 Hz (right). The baseline configuration reproduces the measured oscillation range of the 1:1 demonstrator and serves as the reference for subsequent iterations.

**Figure 14:**
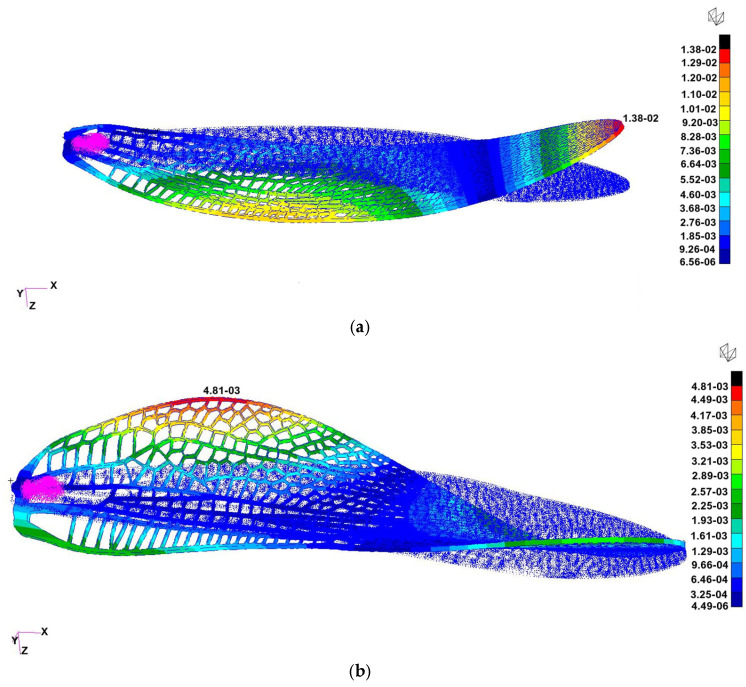
Modal response for Case 4 (Tough 2000 resin, 0.25 mm veins, mass-attached root). The results shown correspond to the 3:1 scaled FEA model, which exhibits (**a**) a first bending mode at ≈130 Hz (free wing with mass attached instead of the support) and (**b**) a torsional mode at ≈334 Hz. For comparison, the 1:1 experimental prototype exhibits a first natural frequency of ≈20 Hz (Section 3.6).

**Figure 15:**
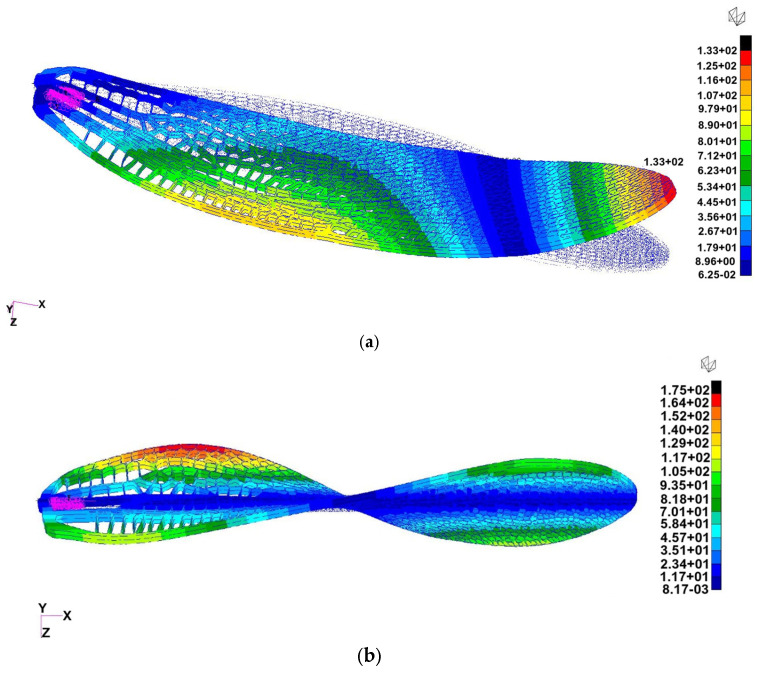
Modal response for Case 5 (Inconel 718, 0.5 mm veins, mass-attached root): (**a**) First bending mode at ≈880 Hz (left), (**b**) first torsional mode at ≈1.8 kHz (right). The metallic configuration illustrates the upper stiffness limit suitable for rigid or hybrid wing skeletons.

Among the six analyzed configurations, Cases 1 (Figure 13), 4 (Figure 14), and 5 (Figure 15) were selected for representation as they encompass the most informative span of the structural parameter space. Case 1 reproduces the physical demonstrator and provides direct experimental correlation; Case 4 demonstrates optimal flexibility for resonance matching; and Case 5 defines the upper stiffness boundary, useful for evaluating hybrid and load-bearing designs. Together, they illustrate the full range of dynamic behaviors achievable through material and geometric tuning.

The modal study confirms that natural frequencies of the printed wing can be effectively tailored by adjusting vein geometry and material selection. Polymeric structures operate within the biologically relevant flapping frequency range, while metallic designs provide structural rigidity and durability. These results validate the design’s scalability and feasibility, offering clear guidance for future iterations that will integrate membranes, actuator coupling, and full aeroelastic simulations.

### 3.4. Preliminary Simulation for the Actuation Mechanism

**Figure 16:**
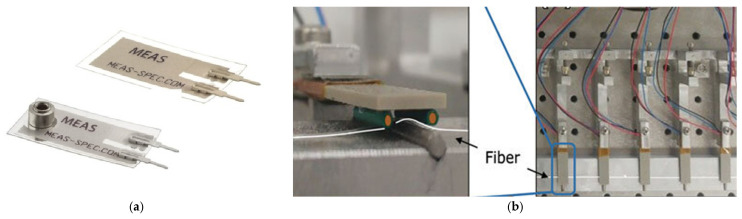
(**a**) PVDF piezoelectric films [53]; (**b**) PZT piezoceramic actuators with fiber details [54].

The first iteration of the actuation concept focused on evaluating piezoelectric bender actuators as the primary driving mechanism for each wing of the dragonfly-inspired vehicle. These actuators are designed to operate under a variable alternating voltage waveform within the desired flapping frequency range (typically 50–100 Hz). To ensure energy-efficient operation, the actuator must be tuned to achieve resonance matching with the wing’s natural frequency. Since piezoelectric devices inherently exhibit higher resonance frequencies, passive tuning strategies—such as length extension through the wing attachment or the addition of tip masses—are introduced to lower the effective resonance toward the desired operating band. Piezoelectric benders operate via the inverse piezoelectric effect, converting electrical energy into precise mechanical displacement. When driven by an AC voltage, the actuator’s free tip oscillates, generating a controlled flapping motion that can be synchronized with the natural structural response of the wing. This direct-drive mechanism eliminates the need for complex gear-based transmissions typically associated with miniature electromechanical systems, thereby reducing mass, improving responsiveness, and increasing overall system agility—critical features for achieving biologically inspired maneuverability. Among available piezoelectric materials, piezoceramic actuators based on Lead Zirconate Titanate (PZT) offer superior performance for the main driving mechanism due to their high piezoelectric coefficient, enabling greater driving forces suitable for sustained flapping under atmospheric pressure. Conversely, PVDF (polyvinylidene fluoride)-based polymer actuators, while less powerful, provide enhanced flexibility and lower density, making them ideal candidates for embedded sensing or hybrid configurations where structural integration and distributed actuation are desired. Figure 16 exemplifies PVDF piezoelectric films (a) and (b) PZT piezoceramic actuators.

To validate the performance of the selected actuation concept, numerical simulations were performed using COMSOL Multiphysics 6.4, coupling Solid Mechanics and Electrostatics through the Piezoelectric Effect module. Both eigenfrequency and frequency-domain studies were conducted on a commercial piezoceramic actuator [55]. The simulated actuator had overall dimensions of 32 × 7.8 × 0.8 mm, composed of two PZT-5A layers (each 0.35 mm thick) with copper electrodes. A driving voltage of 70 V and a harmonic acceleration of 9.81 m/s^2^ (corresponding to 1 g) were applied through a waveform generator, emulating realistic drive conditions at 40 Hz. The actuator used in the simulation is a piezoceramic bender with overall dimensions of 32 × 7.8 × 0.8 mm. It consists of two active layers of PZT-5A piezoceramic material, each with a thickness of 0.35 mm, bonded to copper electrodes that provide the electrical interface. This configuration represents a typical commercial bimorph actuator optimized for low-amplitude, high-frequency deflection suitable for wing excitation experiments. To represent the coupled behavior with the flapping wing, an extension element (40 × 10 × 0.1 mm) was added to the actuator tip, with average material properties corresponding to a carbon-fiber frame with a polymer membrane (density = 1600 kg/m^3^, E = 100 GPa, ν = 0.3, relative permittivity = 5). The combined structure exhibited an eigenfrequency of approximately 106 Hz, whereas the actuator alone had a resonance near 474 Hz, confirming the necessity of passive tuning for frequency matching. By contrast, PVDF-based actuators, due to their lower stiffness, displayed eigenfrequencies around 100 Hz but were unable to sustain comparable loads, reinforcing their suitability primarily for distributed actuation or sensing rather than primary driving.

**Figure 17:**
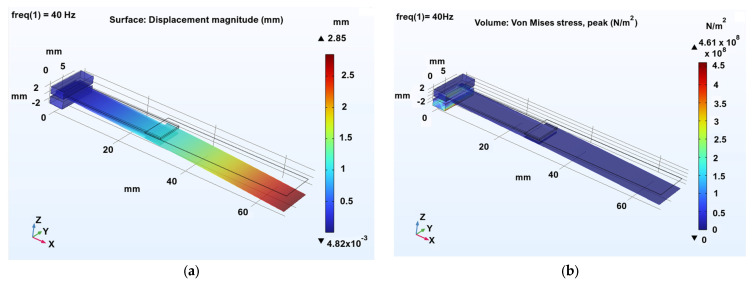
Displacement magnitude (**a**) and von Mises stress (**b**) at 40 Hz.

Figure 17 presents the resulting displacement magnitude and von Mises stress at 40 Hz.

This preliminary analysis demonstrates that PZT-based benders can provide the necessary actuation forces and response characteristics for flapping-wing motion, while PVDF actuators remain viable for lightweight or hybrid concepts. The results serve as the foundation for subsequent experimental validation and design optimization in the following iteration loops.

To increase reproducibility and clarity, additional simulation parameters are provided here. The COMSOL 6.4 model employed a structured mesh with refined elements in the piezoelectric layers to ensure accurate electric–mechanical coupling. A mesh sensitivity analysis showed convergence of the first eigenfrequency within <2%. The piezoceramic layers were modeled using the full piezoelectric constitutive equations under harmonic excitation, with electric potential applied across the copper electrodes. The wing-extension element was assigned orthotropic material properties to reflect the behavior of carbon–polymer laminates. Solver settings included a frequency-domain study with a 0.5 Hz resolution and a direct linear solver for stable convergence. Fixed boundary constraints were applied at the root while all other degrees of freedom remained free.

### 3.5. Ptera Software Results

**Figure 18:**
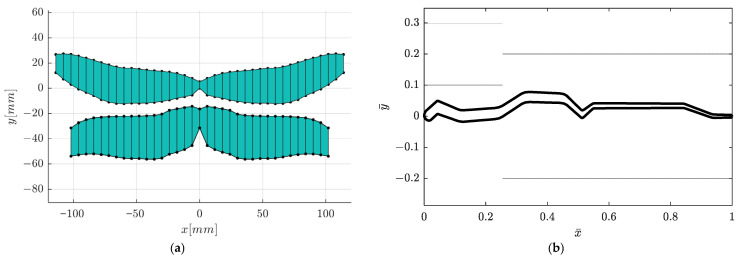
The locations of the leading and trailing edges for both pairs of wings and (**a**) the centroid of the wing ensemble and (**b**) the corrugated profile.

To have a first estimate of the aerodynamic performance of the two pairs of dragonfly wings (both forewings and hindwings), a preliminary simulation of a simplified wing geometry was performed utilizing the Unsteady Vortex Lattice Method, with the help of the Python program Ptera Software 3.2.0 [16]. Figure 18 shows the locations of the leading and trailing edges for both wing pairs, as well as the geometry of the corrugated profile (Figure 17b). The simulation was conducted at an incident air speed of 10 m/s. The simulation captures the motion of the four-wing configuration, consisting of a primary forewing pair and a secondary hindwing pair, as previously mentioned, both executing a sinusoidal flapping cycle.

The visualization of the movement closely resembles the natural flapping motion defined by two primary components: sweeping and pitching, which denote the rotation about the longitudinal and the lateral axes (rolling and pitching axes). The motion is a simple harmonic pattern, with both sweeping and pitching occurring at a high frequency of 40 Hz. The wingtips trace a path dictated by a wide 60-degree sweeping amplitude and a 15-degree pitching amplitude.

A key observation is the phase relationship between the forewings and hindwings. The simulation models an anti-phase flapping pattern, where the hindwings flap exactly π radians (180 degrees) out-of-phase with the forewings. This means that as the forewings move up, the hindwings move down, and vice versa. This counter-stroke motion is a common strategy observed in dragonflies, particularly for achieving high maneuverability, such as hovering or rapid forward acceleration. The geometry utilized in the simulation is constructed from 20 forewing cross-sections and 18 hindwing cross-sections, which discretely define the wing’s airfoil, chord, and leading-edge location. The reference areas and centroids used in this analysis were calculated externally by approximating the wing surface as a series of trapezoids connecting these cross-sections.

**Figure 19:**
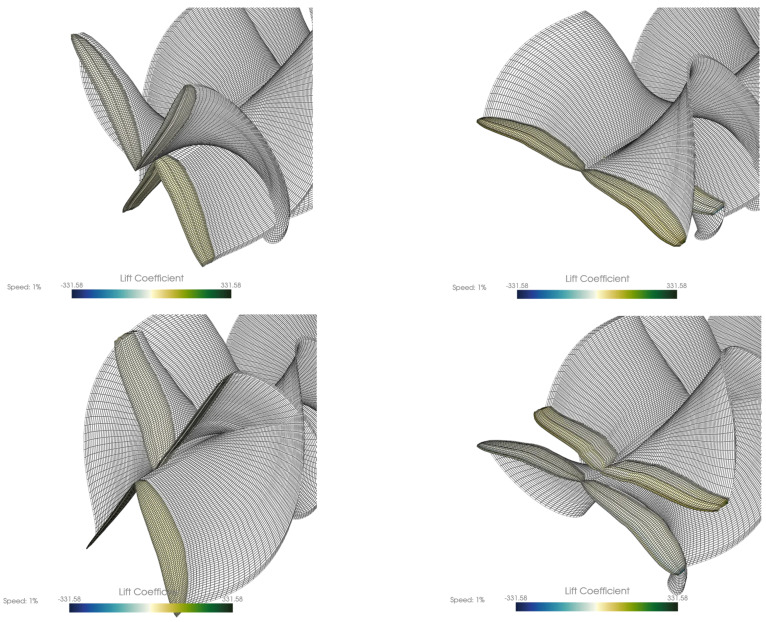
Four stills from one full cycle of the wings resulting from the Ptera Software.

**Figure 20:**
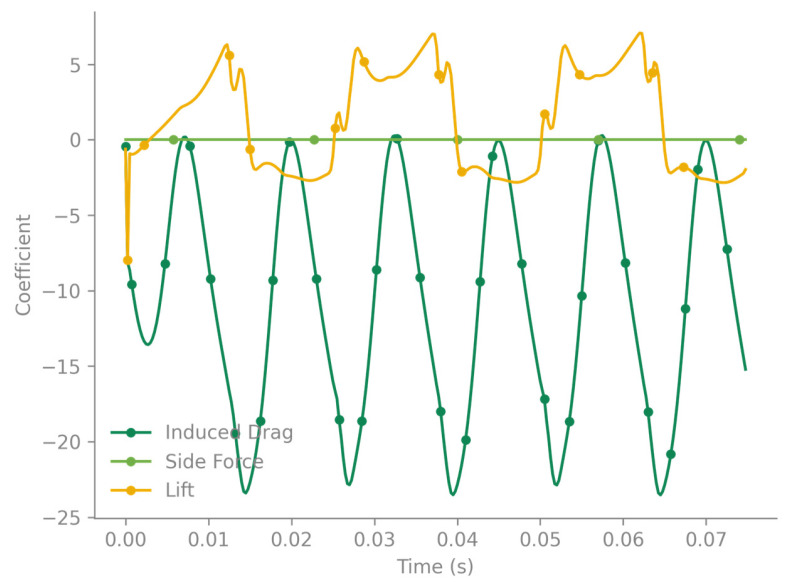
C_L_ and C_D_ evolution.

This geometric simplification is also reflected in the software’s visualization, which generates an approximate mesh by connecting the wing root section directly to the wing tip section (Figure 19). Despite this visual simplification, the underlying 20-section model is crucial, as the wing’s detailed shape directly influences vortex formation. The full animations confirm the complex nature of the aerodynamics across all four wings. Both fore- and hindwings exhibit the same lift generation mechanism, which is operating in anti-phase. The flapping cycle shows moments of intense pressure differential, with strong positive lift (high CL values) generated on the wing’s lower surface during the downstroke and strong negative lift (low CL values) on the upper surface during the upstroke. The lift distribution is consistent with LEV generation, displaying high leading-edge and wingtip suction. The lift is not uniform; rather, it is concentrated near the leading edge and wingtip. Figure 20 presents C_L_ and C_D_ and the total lift and drag forces evolution.

This anti-phase lift generation, where the forewings produce positive lift while the hindwings produce negative lift (and vice versa), likely results in a more continuous, smoothed-out total lift force on the body, reducing oscillations.

The maximum speed of the wingtip (with respect to its body) is $V_{max}=2\pi f\theta_{max}R=30\;m/s$. The maximum total speed is $V_{max,total}=\sqrt{\left(30^{2}+10^{2}\right)}=31.622\;m/s$. The Reynolds number associated with this maximum speed is $Re_{max}=19,452.0$.

### 3.6. Experimental Setups

A preliminary dynamic test was conducted on the 1:1 scale wing prototype to evaluate its natural frequency and the associated unsteady flow behavior. The wing was clamped at the root and subjected to an impulsive deflection by applying a short downward load at the tip, then released to oscillate freely. High-speed imaging at 7500 fps confirmed a dominant oscillation frequency of 24.7 Hz, consistent with the predicted structural mode and in the same range as the biological flapping frequency of dragonfly wings. This test therefore validates both the mass–stiffness scaling of the printed structure and the feasibility of operating near resonance, where the real insect achieves energy-efficient flight.

**Figure 21:**
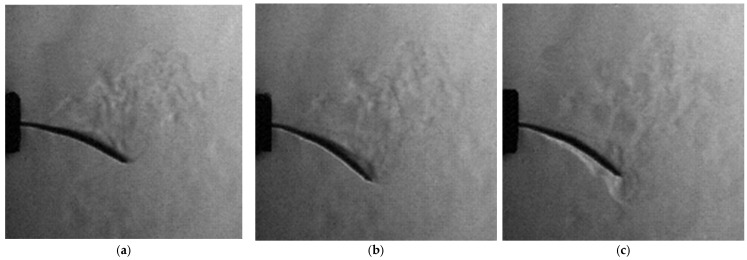
Schlieren image sequence of the 1:1 heated wing oscillating at 24.7 Hz. (**a**) shows a small vortex forming at the wing tip during the initial downward motion, followed by periodic modulation of the surrounding thermal plume, while (**b**,**c**) show the progression of the flow.

The experiment was complemented by classical schlieren visualization, recorded in side view using the Z-type setup. The schlieren sequence presented in Figure 21 captures the thermal and density-gradient field around the heated wing during its oscillation. In the first frame, a distinct vortex structure appears near the wing tip, corresponding to the initial downward motion and local shear-layer roll-up. As the oscillation proceeds, the plume elongates, and the optical contrast alternates with the vibration phase, revealing a periodic modulation of the surrounding flow field. These observations demonstrate that the optical system successfully resolves fine density variations generated by the wing’s unsteady motion, confirming its capability for the heat-dispersion and aeroelastic-coupling analyses foreseen in later phases. To complement the 1:1 dynamic tests, a 3:1 enlarged wing model was evaluated under controlled heating to study thermal propagation and optical visibility at higher spatial resolution. Both forewing and hindwing were observed using simultaneous schlieren and infrared thermography during cooling from 50 °C. The schlieren images revealed distinct density gradients and upward thermal plumes along the leading edge and vein intersections, while infrared maps confirmed localized heat retention in thicker structural regions and faster convective cooling near the trailing edge. These stationary tests demonstrate that thermal and flow behavior is governed by the vein-network topology, validating the fidelity of the printed geometry. The 3:1 configuration improved optical resolution, enabling detailed visualization of convective features and providing a calibration baseline for future dynamic and phase-locked experiments.

**Figure 22:**
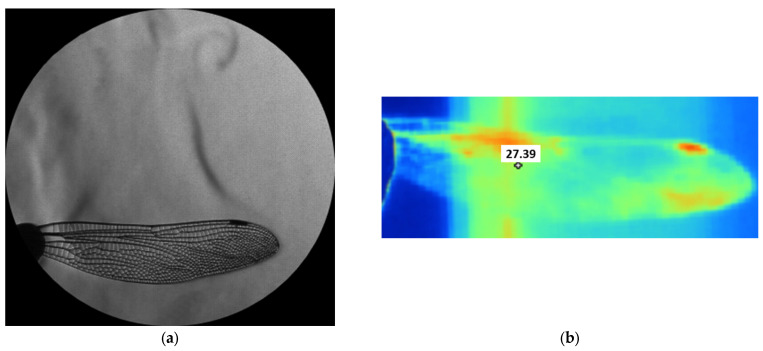

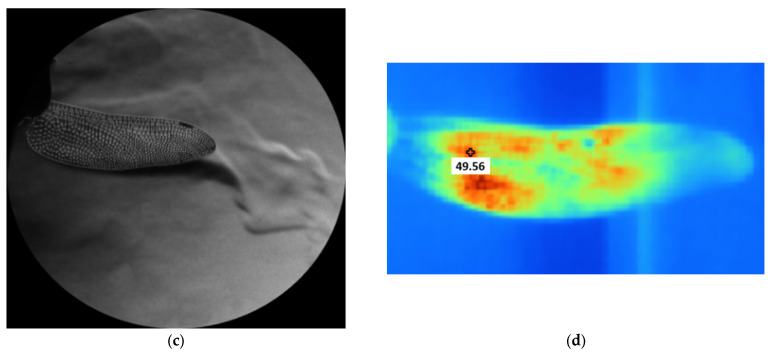
Comparative schlieren and infrared thermography of a 3:1 scale dragonfly wing showing heat dispersion patterns on (**a**,**b**) the forewing and (**c**,**d**) the hindwing.

Figure 22 illustrates the combined schlieren and infrared thermography analysis performed on the 3:1 scale dragonfly wing models, highlighting the differences in heat dispersion between the forewing and hindwing. The schlieren images in panels (a) and (c) capture the thermal plume generated during actuation, revealing distinct flow structures emerging from each wing geometry. These optical signatures correspond closely with the infrared maps in panels (b) and (d), where temperature distributions indicate localized heating along the leading edges and towards the distal regions of the wings. The forewing exhibits a more uniform thermal gradient, while the hindwing shows higher peak temperatures and a broader dispersion zone, reflecting differences in surface area, curvature, and vein density. Together, the schlieren and IR data provide complementary insight into heat transport, aero-thermal interaction, and structural response in the dynamically actuated, bio-inspired wing models.

**Figure 23:**
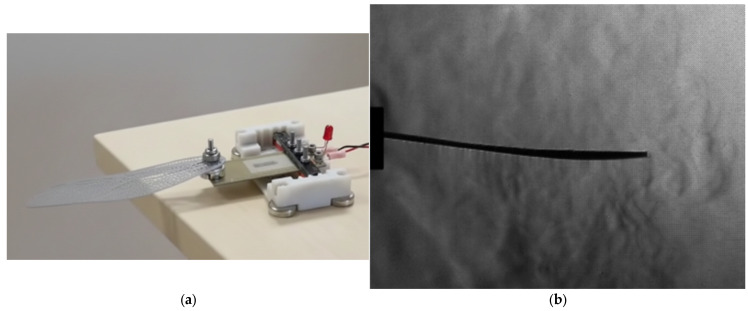
3:1 scale wing dynamic testing: (**a**) setup with compact piezoelectric actuator [54]; (**b**) high-speed frame at 30 Hz (15,000 fps, 1 µs exposure).

The dynamic tests of the 3:1 scale printed wing were carried out using a compact piezoelectric actuator assembly designed for harmonic excitation in the 30–40 Hz range. The actuator was mounted on a custom 3D-printed frame that ensured precise clamping of the wing root and electrical isolation of the driving unit. Powered by a low-voltage signal amplifier, the setup allowed fine control of frequency and amplitude. This configuration (Figure 23a) provided clear optical access for high-speed schlieren, while remaining compatible with future membrane-integrated wings and higher-frequency actuation. Its compact design and open geometry make it suitable for subsequent optical correlation studies and full dynamic characterization of the bio-inspired system.

The tests performed at 30 Hz demonstrated stable, repeatable oscillations corresponding to the first bending mode, with maximum displacement at mid-span and a clear phase lag between root and tip motion. Operating slightly above the measured natural frequency (≈24.7 Hz), the setup produced smooth cyclic deformation with minimal damping, confirming the structural integrity and actuator-wing coupling. These results establish a reliable baseline for upcoming aeroelastic trials with flexible membranes, expected to shift the operating frequency toward the biologically relevant 30–40 Hz range.

## 4. Results

### 4.1. CAD Reconstruction and Bio-Faithful Geometry

The first stage of the results concerns the successful reconstruction of bio-faithful fore- and hindwing geometries derived from high-resolution microscopy and photographic references. The vein topology, nodus placement, and overall structural layout were reproduced with 1:1 dimensional accuracy, enabling a CAD model that preserves the characteristic load-bearing architecture of real dragonfly wings. As shown in Figure 8, the dense, stiffened leading-edge venation and progressively thinning trailing regions were retained in the digital model, ensuring that the printed structures emulate the anisotropic stiffness distribution present in biological wings. Although the first-iteration prototypes were produced with a flat aerodynamic profile, the preserved vein architecture provides the correct mechanical pathways for bending, torsion, and thermal conduction, forming a reliable starting point for structural and aero-elastic evaluation.

### 4.2. Additive Manufacturing Outcomes

The manufacturing process resulted in a high-fidelity 1:1 and 3:1 wing skeleton successfully printed in Tough 2000 resin. As illustrated in Figure 10 and Figure 11, the vein (nervure) network was reproduced with excellent dimensional fidelity, maintaining a uniform 0.5 mm wall thickness across all prototypes. Iterative refinement of printing parameters—layer height, orientation, curing exposure, and drainage channels—eliminated surface defects and ensured repeatability across fabrication cycles. The resulting components exhibited clean vein intersections, well-defined joint regions, and consistent structural rigidity suitable for dynamic testing. Early membrane-integration experiments with chitosan and Kapton^®^ films confirmed compatibility between the printed skeletons and flexible laminates, validating the feasibility of future fully composite wing assemblies.

### 4.3. Structural Dynamics (FEA Modal Analysis)

Finite element analysis demonstrated that the wing’s dynamic response can be effectively tuned through material selection, vein thickness, and boundary conditions. The six simulated cases spanned polymeric and metallic configurations, producing first-mode frequencies ranging from approximately 20 Hz to nearly 900 Hz. This wide range highlights the sensitivity of flapping dynamics to geometric stiffness and inertial loading.

Representative cases—Tough 2000 baseline (Case 1), geometry-softened polymer (Case 4), and stiffened Inconel 718 configuration (Case 5)—are shown in Figure 13, Figure 14 and Figure 15. Case 1 matched the measured oscillation frequency of the physical demonstrator, validating the accuracy of the CAD and print geometry. Case 4 demonstrated that reduced vein thickness combined with root-mass loading shifts the wing toward the biologically relevant 25–30 Hz flapping range. In contrast, Case 5 established the upper stiffness boundary of metallic variants, producing frequencies in the kilohertz range suitable for hybrid load-bearing applications. These results confirm that the printed design offers substantial tunability, enabling future optimization for resonance-matched actuation and aeroelastic functionality.

### 4.4. Actuation Mechanism Simulation

Coupled COMSOL 6.4 simulations of piezoelectric bender actuators provided insight into the feasibility of direct-drive flapping. The commercial PZT-5A bimorph achieved significant deflection under a 70 V harmonic load, with a standalone eigenfrequency of 474 Hz. When coupled to a representative wing-extension element, the resonance decreased to approximately 106 Hz, demonstrating the effectiveness of passive tuning strategies for shifting actuator response toward the operational wing frequency band. The displacement and von Mises stress fields (Figure 17) confirm that the actuator delivers adequate stroke amplitude for driving the first bending mode of the printed wings. Comparative simulations with PVDF actuators showed lower stiffness and load capacity, reinforcing their suitability for hybrid sensing or distributed actuation rather than primary wing excitation.

### 4.5. Aerodynamic Response from Ptera Software

Preliminary unsteady aerodynamic predictions obtained via the Ptera Software vortex-lattice solver reproduced key features of dragonfly-style flapping. The simplified multi-section geometry produced realistic sweeping and pitching kinematics at 40 Hz, with forewings and hindwings flapping in anti-phase. As shown in Figure 18 and Figure 19, this phasing generated alternating positive and negative lift between wing pairs, smoothing the overall force profile across the cycle. These early results confirm that even simplified models capture the essential unsteady mechanisms needed for future higher-fidelity CFD–FSI coupling.

### 4.6. Dynamic Testing of the 1:1 Prototype

Impulse-excitation tests on the printed 1:1 wing validated the numerical predictions. High-speed recordings at 7500 fps confirmed a dominant oscillation frequency of 24.7 Hz, in close agreement with the FEA Case 1 model. Schlieren visualization (Figure 21) revealed distinctive density-gradient structures and vortex signatures near the wingtip during oscillation, demonstrating that the slender printed veins generate measurable aerodynamic perturbations even in simplified setups. The successful capture of these unsteady features verifies the optical sensitivity of the schlieren technique and establishes a foundation for phase-locked measurements in subsequent iterations.

### 4.7. Schlieren and Infrared Analysis of the 3:1 Enlarged Wing

The enlarged 3:1 wing enabled fine-scale visualization of thermo-aerodynamic behavior. As presented in Figure 22, combined schlieren and infrared thermography revealed clear differences in heat dispersion between the forewing and hindwing. The forewing showed a more uniform thermal distribution, whereas the hindwing exhibited higher peak temperatures and broader heat-spreading patterns, reflecting variations in surface area, curvature, and vein density. Schlieren images captured distinct rising plumes and density-gradient features tracing the conduction paths defined by the vein pattern, while infrared maps highlighted areas of heat retention in thicker load-bearing veins. These results demonstrate that thermal transport in the wings is governed by the natural venation architecture, reinforcing the importance of bio-faithful geometry for aero-thermal studies.

### 4.8. Harmonic Actuation of the 3:1 Wing

Dynamic excitation of the 3:1 prototype using a compact PZT-based actuation system (Figure 23) produced stable periodic motion at 30 Hz, corresponding closely to the predicted first bending mode. High-speed schlieren imaging captured the deformation envelope of the wing and confirmed a smooth phase relationship between root and tip motion. The actuator-wing assembly maintained structural integrity and minimal damping throughout the excitation cycle, validating the mechanical coupling strategy. This configuration provides a robust baseline for future tests involving membrane-covered wings, variable actuation waveforms, and resonance-based energy-efficient flapping.

## 5. Discussion

The results of this study highlight the strong potential of bio-inspired venation patterns and structural layouts for small-scale aerial platforms. The reconstructed fore- and hindwing geometries reproduce the anisotropic stiffness distribution observed in natural dragonfly wings, enabling controllable bending and torsion modes that are advantageous for micro air vehicle (MAV) maneuverability. The successful modal tuning into the biologically relevant 20–40 Hz range demonstrates that flapping–resonance coupling is feasible using lightweight polymeric structures and compact actuators. Such resonance-assisted operation is widely recognized as a route toward improving lift generation and reducing energetic cost—two critical requirements for BMAV endurance and operational viability. Unlike previous MAV wing studies that use cartoonized or geometrically simplified wings, the additively manufactured 1:1 model reproduces the exact venation topology and stiffness distribution of the biological wing. The scalable 3:1 model provides, for the first time, an optically accessible surrogate for studying heat dispersion, vortex formation, and deformation modes at large scale. These features collectively advance current biomimetic MAV design by establishing a physically validated, bio-faithful testbed suitable for TRL progression. Comparative FEA simulations reveal that material stiffness and vein-thickness distribution are key determinants of natural frequency, deformation patterns, and actuation compatibility. Polymeric designs (Tough 2000) yield flexible, low-frequency wings suited for resonance-matched piezoelectric actuation, whereas metallic skeletons (Inconel 718) produce high-frequency, stiff structures that would require significantly more energetic input and are more appropriate for hybrid or load-bearing frames. Reducing vein thickness from 0.5 mm to 0.25 mm substantially lowers the modal frequencies, enabling better alignment with typical piezoelectric actuation ranges. Conversely, increases in thickness or stiffness elevate the frequency and reduce wing deformation but can improve structural robustness. These trade-offs emphasize the need for integrative optimization of geometry, materials, and actuation strategy.

Comparative aerodynamic analysis with natural dragonfly wings indicates that the printed prototypes replicate several key biological flight characteristics. Dragonflies typically flap at 25–40 Hz and generate peak lift coefficients in the range CL ≈ 1.2–1.8 during the downstroke, supported by a stable leading-edge vortex extending over 60–70% of the wingspan. Although the present study did not directly measure aerodynamic forces, the 1:1 printed wing exhibited a natural frequency of 24.7 Hz, closely matching the biological flapping regime. Schlieren imaging revealed tip-vortex formation and periodic density-gradient structures qualitatively consistent with LEV onset observed in natural dragonfly hovering. Similar anti-phase lift patterns predicted by the Ptera simulation reflect known forewing–hindwing interactions in Odonata flight. These parallels suggest that the bio-faithful venation architecture of the printed wings captures essential aeroelastic mechanisms despite the absence of membrane camber or dynamic deformation found in real wings.

In the dynamic tests and preliminary Ptera simulations, the anti-phase flapping between fore- and hindwings (180° phase shift) produced alternating positive and negative lift contributions that reduced cyclic fluctuations in total aerodynamic force. This behavior is consistent with known Odonata flight mechanics, where anti-phase motion enhances hover stability by smoothing the instantaneous lift profile and mitigating high-frequency oscillations. Specifically, when the forewings enter the downstroke and generate peak positive lift, the hindwings are in the upstroke, producing a lower-magnitude negative lift that partially cancels the downward force impulse, resulting in a more continuous net-lift curve. Conversely, in-phase motion would synchronize both downstrokes, increasing peak lift but also amplifying force oscillations and structural loading. In the schlieren recordings, this manifests as alternating plume intensities above opposite wing pairs, reflecting the phase-dependent pressure field. These observations confirm that the phase relationship strongly influences aerodynamic stability and lift smoothness, even in simplified dynamic testing conditions.

The criteria introduced in Section 3.2 will guide the membrane selection process in the next design iteration, enabling bio-faithful aeroelastic deformation.

Although the present prototypes were tested under terrestrial atmospheric conditions, the findings provide valuable insight for future Martian applications. Flapping wings achieve force generation through unsteady aerodynamic mechanisms—such as leading-edge vortex stabilization—that remain effective at low Reynolds numbers, which are typical of Mars’ thin atmosphere. However, scaling the wing geometry for Martian flight requires adjustments in wing area, membrane flexibility, and flapping amplitude to compensate for the approximately 1% density relative to Earth. The demonstrated tunability of the polymeric wing structure suggests that mass–stiffness scaling can be adapted for reduced atmospheric loading, while the inherently lightweight venation architecture is well aligned with the mass constraints of planetary micro-explorers. The present work therefore contributes foundational knowledge needed to explore BMAV feasibility for Mars scouting missions.

A key outcome of this study is the clear demonstration that bio-faithful venation networks offer mechanical and aero-thermal advantages over simplified planar wings. The vein topology not only governs bending and torsional modes but also influences heat dispersion and convective behavior, as shown by the schlieren and IR results. In contrast, simplified wings lacking structural heterogeneity would not exhibit the same conduction patterns or anisotropic deformation modes. Preserving biologically accurate geometry therefore supports more realistic aeroelastic coupling, enhances interaction with natural vortex structures, and improves predictive fidelity for MAV design. The combined numerical and experimental evidence underscores that true-to-nature wing structures provide more relevant insights for design iteration than idealized models.

Despite these advantages, the present demonstrator has several limitations. First, the printed wings were produced with flat profiles, omitting the natural camber and corrugation that significantly affect aerodynamic performance. Second, the wings lacked a membrane layer during the initial mechanical and thermal tests, limiting the ability to replicate natural aeroelastic responses. Third, the simplified actuation system operated at a single frequency band and did not replicate full multi-axis dragonfly flapping kinematics. Fourth, the aerodynamic simulations (Ptera) employed simplified geometries and quasi-steady vortex-lattice assumptions that cannot capture 3D viscous effects. Finally, testing was performed under static or sinusoidal forcing, without phase-locked measurements or integrated flight-like conditions. These constraints define clear boundaries for interpreting the results.

To advance the system toward higher technology readiness levels (TRL), several steps are recommended. Incorporating flexible membranes into the printed venation network will enable realistic aeroelastic deformation and more accurate aerodynamic performance. Introducing corrugation or mild camber into the CAD model will improve LEV stability and bring the geometry closer to true dragonfly wing profiles. Integrating a multi-degree-of-freedom actuation system, capable of independently modulating sweeping, pitching, and stroke-plane angle, will allow evaluation of biologically relevant kinematic modes. Higher-fidelity CFD–FSI simulations should replace simplified vortex-lattice methods to resolve vortex formation and stability across all wings. Finally, controlled wind-tunnel tests and in-flight bench demonstrations would provide the necessary validation for TRL progression toward functional BMAV prototypes. Collectively, these improvements will transform the current structural demonstrator into a fully coupled, bio-inspired flapping-wing system ready for advanced aeroelastic and planetary-flight investigations.

## 6. Conclusions

This work presented the first iteration of a bio-faithful dragonfly-inspired flapping-wing system, integrating realistic venation geometry, additive manufacturing, structural modeling, and preliminary aerodynamic and thermo-optical testing. The CAD reconstruction successfully preserved the natural vein topology, enabling 3D-printed prototypes that reproduced the anisotropic stiffness patterns of real wings. Modal analysis and impulse testing confirmed that polymeric designs operate within biologically relevant flapping frequency ranges, while material and geometric adjustments offer effective tuning of structural response. Simplified aerodynamic simulations captured the essential anti-phase lift dynamics of four-wing flapping, and schlieren/infrared experiments demonstrated that heat dispersion and flow features follow the vein-driven structural pathways of the printed wings.

Overall, the results validate the feasibility of using bio-inspired structural layouts for MAV development and establish a solid foundation for future iterations that will incorporate membranes, full kinematic actuation, and higher-fidelity aeroelastic modeling.

The results of this study offer several insights for future full-scale biomimetic MAV design. First, the demonstrated ability to tune wing natural frequencies into the biologically relevant 20–40 Hz range indicates compatibility with lightweight piezoelectric actuators commonly used in miniature flapping systems. Second, the bio-faithful venation network provides a structurally resilient architecture capable of distributing loads and thermal stresses, suggesting that similar skeleton–membrane composites could endure repeated flapping cycles with reduced risk of fatigue. Finally, the scalability of the printed wings, combined with their passive aerodynamic mechanisms, highlights potential applicability in low-density extraterrestrial atmospheres such as Mars, where unsteady lift generation is essential for flight at low Reynolds numbers. These findings collectively support the feasibility of extending bio-inspired, venated-wing concepts toward operational MAV platforms.

Looking forward, the results of this first-iteration demonstrator establish a clear foundation for developing a fully functional flapping-wing MAV based on a bio-faithful venation architecture. Future work will integrate compliant membranes to enable realistic aeroelastic deformation, implement multi-degree-of-freedom actuation for controlled sweeping and pitching, and employ high-fidelity FSI simulations to resolve vortex formation and aerodynamic loads. The scalable design methodology demonstrated here also opens pathways for lightweight planetary micro-aerial platforms, including Martian flight concepts where unsteady, low-Reynolds-number aerodynamics are required. Overall, this work provides a validated structural and manufacturing baseline from which higher-TRL prototypes—capable of stable hovering, maneuvering, and autonomous flight—can be developed.

## Data Availability

All generated data is contained in the article or can be made available upon request.
